# Levothyroxine and Prednisone Causing Generalized Weakness in a Middle-Aged Man

**DOI:** 10.1155/2012/616930

**Published:** 2012-10-18

**Authors:** Andrew Word, Kevin Davidson, Essam Elsayed

**Affiliations:** ^1^Departement of Internal Medicine, UT Southwestern Medical School, 5323 Harry Hines Blvd., Dallas, TX 75390, USA; ^2^Division of Nephrology, Dallas VA Medical Center, 4500 S Lancaster Road, Dallas, TX 75216, USA

## Abstract

Thyrotoxic induced hypokalemic periodic paralysis is a rare disorder that had been described in middle-aged men, predominantly Asians and Hispanics. This case presented with generalized weakness and hypokalemia after changing prescription for levothyroxine and starting prednisone to treat upper respiratory infection in a previously asymptomatic middle-aged Hispanic male. In this paper, we will go over the clinical presentation, mechanisms, and treatment of thyrotoxic induced hypokalemic periodic paralysis. Our objectives are to identify the classic constellation of findings in thyrotoxic periodic paralysis and to recognize the importance of considering thyrotoxic periodic paralysis among patients with hypokalemia.

## 1. Case Presentation 

A 40-year-old Hispanic gentleman with a history significant for Graves' disease with prior radioablation treatment fifteen years ago presented with generalized weakness of his trunk and extremities. The generalized weakness began three days earlier when the patient “overindulged on carbohydrates and alcohol.” The patient was unable to exert himself to his baseline and had experienced two falls since the onset of symptoms. Of note, he was treated for sinusitis and bronchitis four days earlier. The patient admitted to tremors, palpitations, heat intolerance, diaphoresis, and diarrhea. He denied fever, chills, weight loss, shortness of breath, and polyuria. The patient admitted to being a smoker in the past, occasional alcohol use, and denied illicit drug use. The patient was currently taking levothyroxine 300 mcg/day, metoprolol 200 mg/day, and hydrochlorothiazide 50 mg/day. The sinusitis was being treated with prednisone 60 mg/day and Augmentin 250 mg/day. 

Physical exam revealed a tremulous male with no proptosis, thyromegaly, tachycardia, or pretibial myxedema. However, the patient's skin was notably flushed and diaphoretic on his neck, trunk, and palms. His neurologic exam revealed 4/5 motor strength throughout. The patient exhibited truncal weakness as he had to help himself to a sitting position by using the bed's sideboards and was unable to sit without assistance for more than a few minutes. 

Laboratory analysis showed a decreased potassium of 2.4 mEq/L (normal of 3.5–5 mEq/L), glucose of 174 mg/dL, TSH < 0.1 mIU/L (6 months ago was 1.1 uIU/mL), and free T4 of 1.81 ng/dL (normal of 0.8–1.5 ng/dL). Subsequently, the patient was administered 40 mEq oral potassium chloride. Prednisone was discontinued as was hydrochlorothiazide until resolution of hypokalemia. Metoprolol was continued, and levothyroxine was temporarily held. The patient's symptoms progressively resolved within hours. He was discharged home the next day on a decreased dose of levothyroxine.

## 2. Discussion

Patient's symptoms were diagnosed as being secondary to thyrotoxic periodic paralysis (TPP). Despite the rare incidence of thyrotoxic periodic paralysis, this disorder represents an easily recognized and reversible form of hypokalemic-mediated generalized weakness in patients with hyperthyroidism. The above case exhibits such a situation and therefore has direct relevance to the general internist. In this case, the classic findings of TPP, weakness with associated thyrotoxicosis and hypokalemia, are depicted. TPP occurs sporadically, showing no familial inheritance pattern as is seen with the autosomal dominant inheritance of familial hypokalemic periodic paralysis. It has been proposed that patients with TPP have an ion channel defect that is exacerbated in the face of thyrotoxicosis but not in a euthyroid state [1]. Thyroid hormone increases tissue responsiveness to *β*-adrenergic stimulation by increasing the activity of Na-K ATPase pumps, which results in an intracellular shift of potassium. This scenario is exacerbated in the setting of strenuous exercise, high-carbohydrate load, hyperinsulinemia, or any event inciting an elevated stress response, including cold exposure, infection, alcohol intake, and corticosteroid administration [1–3]. 

Corticosteroids have been shown to stimulate insulin secretion and to increase the number of Na-K ATPase pumps, both of which increase the likelihood of intracellular potassium shift [4]. Pertinent to this discussion, generalized weakness in the above case is present in a patient with laboratory-confirmed thyrotoxicosis and hypokalemia in the setting of maximum levothyroxine dose, glucocorticoid treatment, and recent high-carbohydrate load. 

The patient's very low TSH was related to a change in the supplier of his levothyroxine from a local pharmacy to an online pharmacy and despite no change in strength in the 9 months prior to presentation but change of generic was previously reported to affect strength of some drugs such as levothyroxine and that might explain the thyrotoxic picture [5, 6]. The American Thyroid Association recommends that patients should be maintained on 1 brand of levothyroxine product and serum TSH levels should be checked 6 weeks after switching a patient from one brand to another brand [7]. Another explanation is that the patient might became more adherent in taking his medications after recent illness or an increase in absorption of levothyroxine with changes in diet. We found no reported interactions between prednisone and levothyroxine. 

Thyrotoxic periodic paralysis is a rarely seen condition in patients with hyperthyroidism, but identification of its pathognomonic presentation in a thyrotoxic patient can significantly reduce the risk of developing life-threatening complications associated with hypokalemia, such as respiratory weakness and arrhythmias. Treatment consists of correction of underlying thyrotoxicosis with PTU, methimazole, radioablation, or correction of levothyroxine levels. 

Potassium levels are corrected with potassium chloride, and *β*-blockers can be initiated to prevent continued intracellular potassium shifts. It is important to note that we should avoid repleting potassium using IV solutions that has glucose such as D5W as that can exacerbate the hypokalemia from stimulating insulin secretin with subsequent further shift of the potassium to inside the cells. Other contributing factors, such as corticosteroid use, must also be monitored. Medical alert bracelets are strongly recommended for TPP patients. In our case, thyrotoxicosis was likely iatrogenically-induced, so the levothyroxine dose was lowered. The corticosteroid was also discontinued because of its contribution to hypokalemia. As exhibited in this case, internists should be vigilant to consider TPP as a cause of generalized weakness in thyrotoxic patients. In such cases, the internist should be cognizant of the importance of correction of contributing factors, such as appropriate titration of levothyroxine and corticosteroid dosages.
